# Barriers and facilitators to dissemination and adoption of precision medicine among Hispanics/Latinos

**DOI:** 10.1186/s12889-020-08718-1

**Published:** 2020-05-01

**Authors:** Juan R. Canedo, Consuelo H. Wilkins, Nicole Senft, Araceli Romero, Kemberlee Bonnet, David Schlundt

**Affiliations:** 1 School of Graduate Studies and Research, Meharry Medical College, Nashville, TN USA; 2 Vanderbilt University Medical Center, Nashville, TN USA; 3 Meharry-Vanderbilt Alliance, Nashville, TN USA; 4 Progreso Community Center, Nashville, TN USA; 5 Department of Psychology, Vanderbilt University, Nashville, TN USA

**Keywords:** Precision medicine, Barriers, Facilitators, Knowledge, Awareness, Disparities, Dissemination, Adoption, Hispanics, Latinos

## Abstract

**Background:**

With the rapid advances in gene technologies in recent years, the potential benefits of precision medicine (PM) may spread unevenly to disadvantaged populations, such as Hispanics/Latinos.

The objective of this study was to explore patient-level barriers and facilitators to dissemination and adoption of PM among Hispanics/Latinos, including knowledge and awareness.

**Methods:**

Self-identified Hispanics/Latinos from diverse countries in Latin America (*N* = 41) participated in the study. Using a cross-sectional observational qualitative research design, six focus groups and a demographic questionnaire were collected in English and Spanish. Qualitative content analysis was utilized to code the transcripts and identify emerging themes.

**Results:**

Hispanics/Latinos never heard of and had no knowledge about PM. Barriers to dissemination and adoption of PM included lack of health insurance, financial burden, participants’ immigration status, distrust of government, limited English proficiency, low literacy levels, cultural norms, fear about genetic testing results, lack of transportation, newness of PM, and lack of information about PM. Facilitators included family support; information provided in Spanish; use of plain language and graphics; assistance programs for uninsured; trust in physicians, healthcare staff, well-known hospitals, academic institutions, and health care providers and community organization as sources of reliable information; personal motivation, and altruism or societal benefit.

**Conclusions:**

Culturally-and linguistically-tailored, low-literacy educational material about PM should be created in English and Spanish. Future research should examine provider-level and system-level barriers and facilitators to implementation and adoption of PM among Hispanic/Latino patients.

## Background

Precision medicine (PM) has changed the traditional one-size-fits-all approach to healthcare by providing tools for physicians to consider patients’ individual genetic makeups, environments, and lifestyles for the diagnosis, prevention, and treatment of diseases [1–3]. Advances in gene technologies have improved the ability for PM to lead to reductions in disease burden and mortality. However, at the same time, breakthroughs in PM have the potential to widen health disparities. The dissemination of innovations in healthcare tends to be slow and uneven, benefiting socially advantaged groups more quickly than disadvantaged groups, such as racial and ethnic minorities, low income populations, or uninsured patients [4]. The continued diffusion of PM innovations into healthcare practice is influenced by many factors, including the willingness of patients to adopt new PM approaches, particularly in relation to the use of their genetic information [5]. For example, when healthcare providers offer genetic testing to patient to determine their risk for a disease or pharmacogenomic testing to guide the selection and dosing of medications, patients must decide if they consent to the testing and if they want to know the results. Patient acceptance of using a PM approach as part of their healthcare could be limited by numerous barriers, such us lack of knowledge and cost, which may limit patients’ motivation or ability to seek, obtain, and make informed PM healthcare decisions [6–8]. However, acceptance and uptake could also be facilitated by factors such as trust in their doctors and altruistic motivations to contribute to scientific knowledge.

Awareness of the term “precision medicine” among adults in the US is low, estimated at less than 25% in a recent study [9], even though approximately 80% of adults in the U.S. report that they have heard of genetic tests, which are a key component of PM [10]. A recent systematic review of studies published through 2017 [11] identified no studies that compared racial/ethnic differences in awareness of PM, though one study found no difference between Blacks and Whites in awareness of the related term “personalized medicine” [12]. This review also found Blacks and Hispanics/Latinos had lower factual knowledge about PM and more concerns about genetic testing compared to Whites.

Knowledge and attitudes towards PM are especially understudied among Hispanics and Latinos, terms we use interchangeably to refer to persons from Latin American countries whose primary language is Spanish [13]. Diffusion of Innovations Theory [14], which take into account the perception of the innovation, characteristics of people who adopt or fail to adopt the innovation, and the contextual factors that determine the adoption or lack of thereof [5]. Informed by this theory, the aim of this study was to explore patient-level barriers and facilitators to disseminating and adopting of PM approaches as the innovation in healthcare among Hispanic/Latino adults, including knowledge and awareness of PM. The findings can be used to inform the development of patient education materials to address identified barriers and facilitators to increase dissemination and adoption of PM approaches among Hispanic/Latino patients.

## Methods

### Study design

A cross-sectional observational qualitative research design was used to facilitate organized focus group discussions. Participants’ interactions were the source of qualitative research data to explore patient-level barriers and facilitators to dissemination and adoption of PM [15–17]. This study used the community-engaged research (CEnR) approach facilitated by a well-established community-academic partnership between Progreso Community Center (PCC) and Meharry Medical College (MMC). PCC is a Hispanic/Latino nonprofit community-based organization in Nashville, Tennessee that shares MMC’s interest in exploring the dissemination of PM as an innovation in healthcare among Hispanics/Latinos in Nashville [14, 18, 19]. Community partners at PCC collaborated with the academic partner at MMC from conceptualization through dissemination. They contributed to developing the questionnaire and the focus groups discussion guide and translating these from English to Spanish. They also jointly participating in analyzing and interpreting the qualitative data and contributed to dissemination of findings. The academic partner at MMC led the research design and oversaw the data collection and data analysis.

The Meharry Medical College Institutional Review Board approved this study including verbal consent (waiver of documentation of consent) to participate in it because the study presented no more than minimal risk of harm to subjects. Both MMC and PCC staff obtained their human subjects research and compliance certificates from the Collaborative Institutional Training Initiative (CITI Program).

### Characteristics of participants and recruitment

The study was conducted in Nashville, Tennessee, which is considered a “new-growth community” due to the rapid increase of Hispanic/Latino immigrants in recent decades with low English proficiency and cultural diversity [20]. Approximately 10% of the population in Nashville/Davidson County consists of Hispanics/Latinos [21]. While Mexicans represent the largest group of Hispanic/Latino immigrants in Nashville, this community is heterogeneous in countries of origin [22]. Therefore, participants from diverse Latin American countries of origin in addition to Mexico were recruited for the study. The inclusion criteria were adults ages 18 and older who self-identified as Hispanic or Latino living in the Nashville metropolitan area. There were no exclusion criteria.

Convenience sampling was used to recruit potential participants by telephone using PCC’s members list, making announcements at in-person PCC activities, and distributing fliers in local organizations and business that cater to Hispanics/Latinos. Interested persons could choose to participate in a focus group conducted in Spanish or in English. PCC staff attempted to recruit approximately 10–12 people to attend each focus group. Data collection and analysis were conducted iteratively in order to stop recruitment when thematic saturation was reached, i.e., when no new themes or information emerged from new groups.

### Data collection instruments

The academic and community partners developed the focus groups discussion guide based on the Diffusion of Innovations Theory [14], with questions covering the following topics: knowledge and awareness of genes, genetics, PM, barriers and facilitators to dissemination and adoption of PM. They also created a demographic questionnaire based on previous studies conducted by the partners that included questions about educational attainment, income, health insurance coverage, English proficiency, self-reported health status, and first and second-degree family health history. PCC staff translated the English versions of the questionnaire and the focus groups discussion guide to Spanish. Then the translations were reviewed a committee of six Spanish-speaking Hispanic/Latino community members from Mexico, Central, and South America to give feedback on the Spanish translations to make sure the words were clear and would be interpreted the same way across focus groups participants from various Latin American countries. The research team revised the questions based on their feedback. The individuals who participated in the translation process were not invited to participate in the focus groups to avoid participants’ contamination.

### Data collection

All participants provided verbal informed consent in English or Spanish, based on language of preference, before completing the survey and participating in the focus groups. Each participant was assigned an ID number to ensure anonymity and confidentiality. After informed consent, participants completed a questionnaire in Spanish or English, and participated in a focus group. Focus groups were facilitated by the academic partner either in English or Spanish, and PCC staff took notes during the focus groups sessions, which were also audio recorded. Participants received a $30 gift card at the end of each focus group.

**Fig. 1:**
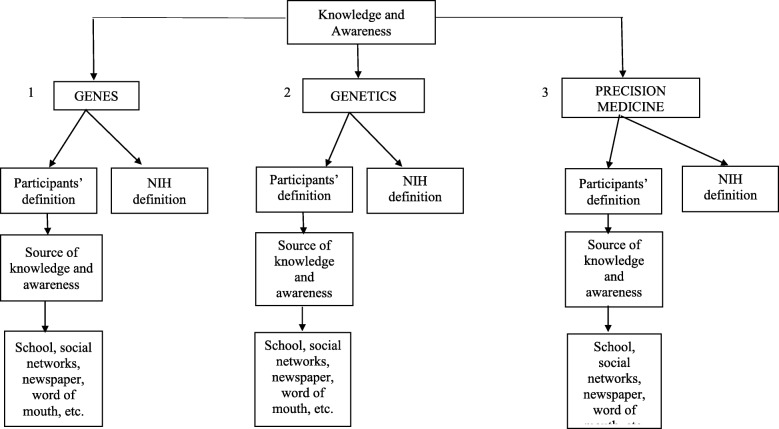
Flow of Question about Knowledge and Awareness of Genes, Genetics, and Precision Medicine

In each focus group, first the facilitator asked participants about their knowledge or awareness of the terms genes, genetics, and PM. For each term, after participants’ discussion about their own knowledge and awareness of the term, the focus group facilitator read out loud the definition of the term from the National Institutes of Health (NIH) and showed it written on a flip chart (Fig. 1). Next, participants reacted to the NIH definition for each term by discussing similarities or differences between NIH definitions and the definitions they had provided. After repeating this process for each of the three terms, the facilitator asked questions about patient-level barriers and facilitators to dissemination and adoption of PM.

### Data analysis

The focus groups were audio recorded and transcribed verbatim by bilingual PCC staff in the language in which the group was conducted, either English or Spanish. NVivo qualitative software version 11.4.3 for Mac was used to analyze the data. The academic partner trained one member of the PCC staff to use NVivo to assist with analyzing the qualitative data. Qualitative content analysis was used to organize and summarize key themes from manifest analysis, which included the transcription of what participants exactly said in the focus groups to latent analysis for the interpretation of the meaning contained in the text of each transcription [23]. Codes were created for both languages with references and corresponding a priori codes about specific themes based on the discussion guide questions, and new nodes were created for emerging themes during analysis.

First, the academic partner and community partner each reviewed the transcripts and created codes for each focus group independently. Subsequently, the two partners reviewed their coding for each group together to manage, compare, and discuss emerging themes, or modify the coding scheme as needed. This process was repeated for each group until reaching thematic saturation. Overall, the two partners had 80% agreement in coding, and they resolved all of the differences through discussion to reach consensus [24]. Additionally, for the demographic questionnaire, descriptive quantitative data analysis was conducted using SPSS version 25.

## Results

In total, 41 participants took part in six focus groups (four in Spanish and two in English), with 3 to 7 participants attending each group. The number of attendees was lower than recruited due to no shows. Close to two thirds (60%) of participants were female, the majority of participants 44% were from Mexico; 17% from El Salvador; 7% from Honduras; 5% each from Bolivia, Chile, and Venezuela; 2% from each Guatemala, Nicaragua, and Colombia; and 10% native born in the U.S. Married participants were 43%, single-never married 38%, divorced 5, and 15% living with a partner. Over a third of participants (34%) completed 8 years or less of school, 49% completed high school, and 17% had post high school education. Over two thirds (68%) of participants had a monthly income less than $3000. Almost half of participants (45%) had lived less than 10 years in the U.S. And over half of them (55%) had lived more than 10 years in the U.S. Half of participants (54%) self-reported knowing either nothing or a little of the English language. The majority of participants (93%) were younger than 50 years of age. See (Table 1).

**Table 1:** Demographic Characteristics of Participants

	N	%
**Age**		
18–29	16	39.0
30–49	22	53.7
50–70	3	7.3
**Gender**		
Male	15	40.0
Female	24	60.0
**Insurance**		
Insured	5	12.8
Uninsured	32	82.0
Medicaid (TennCare)	1	2.6
Medicare	1	2.6
**Country**		
Mexico	18	43.9
El Salvador	7	17.1
Honduras	3	7.3
Guatemala	1	2.4
Nicaragua	1	2.4
Bolivia	2	4.9
Colombia	1	2.4
Venezuela	2	4.9
Chile	2	4.9
United States	4	9.8
**Marital Status**		
Married	17	42.5
Single-never married	15	37.5
Divorced	2	5.0
Living with partner	6	15.0
**Years of education**		
0–5	1	2.4
6–8	13	31.7
9–12	20	48.8
13–17	7	17.1
**Years living in the U.S**.		
1–4	15	36.7
5–9	2	4.9
10–14	6	14.6
15–19	6	14.6
20–24	6	14.6
25–30	6	14.6
**Monthly income**		
Less than 2000	11	28.9
2000-2999	15	39.5
3000-2999	5	13.2
4000-4999	1	2.6
5000-5999	1	2.6
6000 or more	3	7.9
10,000 or more	2	5.3
**English proficiency**		
Excellent	8	19.5
Very good	5	12.3
Good	6	14.6
Little	16	39.0
Nothing	6	14.6
**Employment**		
Full time (32 + hrs week)	22	55.0
Part time (less than 32 h week)	5	12.5
Unemployed	6	15.0
Home maker	5	12.5
Other	2	5.0

### Knowledge and awareness of genes

Before introducing the NIH definition of genes, the majority of participants knew that genes are related to human heredity and are passed from generation to generation to determine specific traits and characters in people, such as height, hair color, eye color, personality, and diseases (See sample quotes in Table 2). Most participants had a general knowledge about what genes were; as a participant mentioned, “I understand that genes are a certain type of components that are inherited from generation to generation;” however, some participants had no or incorrect knowledge about genes. For example, one participant said, “[f] or me, they [genes] are the cells that form men’s red and blue blood.”

**Table 2:** Awareness and Knowledge about Genes, Genetics, and Precision Medicine

Genes		
NIH Definition *“A gene is the basic physical and functional unit of heredity.”*	Awareness • “I heard about genes in high school and university.” • “… in high school in my genetic class.” • “I heard about it in school.” • “My grandmother used to tell us that diseases are inherited from relatives … She had cancer, and she was really worried that we could inherit cancer from her.” • “My mother told me that I have my father’s genes because I am moody like he is.” • “I read about genes in the newspaper and the internet.”	Knowledge • “I understand that genes are certain type of components that are inherited from generation to generation … I learned about them in high school.” • “Genes are like in the blood. For example, if someone is moody, people tell you that you have your father‘s genes. [Genes] are something that are inherited from family.” • “For me, genes are when you inherit something from your father, mother... you inherit a disease or a similar trait.” • I had heard for example, if I have diabetes o some disease, and I am going to have a baby, like the disease can be transmited.
Genetics		
NIH Definition *“Genetics refers to the study of heredity and changes in inherited characteristics.”*	Awareness • “I heard about genetics in college in a biology course.” • “I never heard about it in school. I remember that my teacher talked about genes, no genetics.” • “I heard my relative saying that genetics and genes are the same.” • “I read about it on the internet, but I did not understand.” • “I never heard about genetics anywhere.”	Knowledge • “I always heard that human genetics is like the formation of the beginning of the world, it might be that is what differentiates animals from human beings.” • “Honestly, I do not have much idea about genetics. I have lipomas, and do not know if that is because of genetics or genes …” • “For me, genetics it the science that improves, alters, or study genes. I think that is the blood tie that develops the biological watch throughout time in a human being and. Differentiates different negative or positive aspects in a human being and in animals.” • “I understand that genetics is a science that studies genetics [and] genes.” • “I do not know what genetics is.”
Precision Medicine		
NIH Definition “*Precision medicine refers to a new approach for disease treatment and prevention that takes into account individual difference in genes, environment, and lifestyle for each person.”*	Awareness • “I have never heard anything about it.” • “I had not ever heard about it until now that you mentioned it. I had never heard that term.” • “I never heard about that type of medicine.” • “I had the least idea about precision medicine, the term, never had heard about it.”	Knowledge • “I understand that definition. It seems clear to me, but it still sounds like science fiction. It would be something unbelievable that medicine’s approach a person could be cured at the first visit to the doctor’s office.”

When the focus group facilitator showed and read out loud the National Institutes of Health (NIH) definition of genes, participants were gladly surprised that their own definitions of genes were somewhat similar to the NIH definition; however, participants mentioned that terms used by the NIH’s in its short definition used technical and hard to understand words (See Table 2). Consequently, they mentioned that the definition may not be understood by Hispanics/Latinos if they have low educational attainment and did not already know about genes. None of the focus group participants mentioned that doctors or other healthcare providers had explained to them what genes are; furthermore, they did not mention healthcare settings, such as doctors’ office, hospitals, or community clinics as places where they heard or learned about genes*.*

### Knowledge and awareness of genetics

Following the same steps across all focus groups, participants were asked about their knowledge or awareness of genetics. Before seeing the NIH definition, the majority of participants did not know that genetics refers to the study of heredity and the changes that occur in inherited characteristics. Some definitions were partially true, for example, one participant mentioned, “I learned in college that genetics is the science that improves, alters, or studies genes.” Others showed little or no knowledge of genetics. For example, one participant said, “I think that genetics is the symmetry that studies the body.” The majority of participants shared that their knowledge and awareness of genetics was not through school, but through personal social networks, traditional media, and social media. During the discussions, the majority of them defined genetics interchangeably as genes; for example, “I heard that people say that sometimes when a child is mischievous, it is because of the family genetics and carries [that behavior] in the genes.” Although participants knew that there were no right or wrong answers in the focus group discussions, they were disappointed that their genetics definition was not as close as the NIH definition, and mentioned that the definition needed to be clarified and adapted to better communicate what genetics is. Once more, none of the focus group participants shared that physicians or healthcare staff explained genetics to them; furthermore, they did not mention healthcare settings as places where they heard or learned about genetics*.*

### Knowledge and awareness of precision medicine

Finally, the focus group facilitator asked participants about their knowledge and awareness of PM to continue the conversation about barriers and facilitators to dissemination and adoption of this new approach in medicine. Across all focus groups in English and Spanish, no participants knew about or had ever heard about PM (See Fig. 1). The focus group facilitator proceeded to show and read out loud the NIH’s definition of PM. After hearing the definition, some participants said, “I never heard about it” and “I had no idea about the term precision medicine, I never heard about it.” After participants read and heard the NIH definition of PM, the majority of them were confused by the term “environment” because they associated the term only with the natural environment, such as air, rain forests, lakes, rivers etc. After discussing the term more, they suggested that educational materials should use pictures to clarify the broader meaning of the word “environment” in this context to include the built environment and social determinants of health, such as the presence or absence of infrastructure in the geographic areas where people live, including basic services, schools, grocery stores, parks and hospitals infrastructures.

### Barriers to dissemination and adoption of precision medicine approaches

**Fig. 2:**
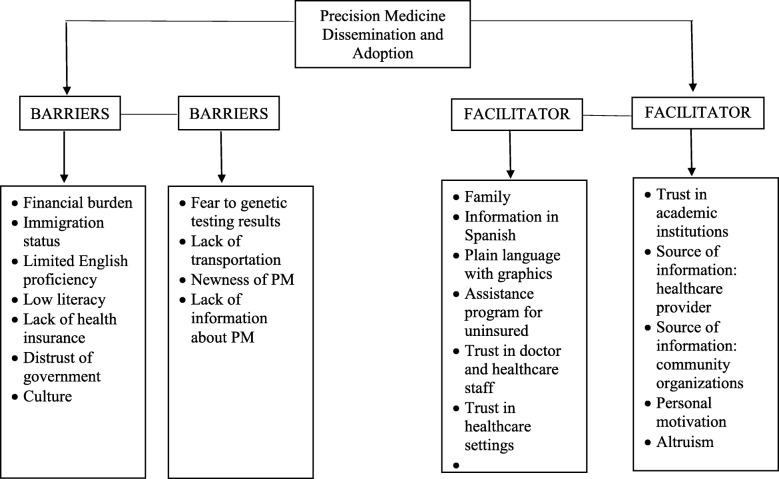
Barriers and Facilitators to Dissemination and Adoption of Precision Medicine

Figure 2 lists the barriers to dissemination and adoption of PM approaches that emerged as themes during the focus groups, and Table 3 provides representative quotes for each theme. Among the main barriers mentioned by participants were the lack of health insurance and the potential financial burden of PM due to the cost of genetic testing and treatment. Immigration status was another barrier to the dissemination and adoption of PM mentioned by participants. Participants referred to immigration status as a factor that determines whether they will seek care in healthcare settings. If they decide not to seek care due to their undocumented immigration status, they will not have access to information about PM in healthcare settings. If they do not know what PM is and are not offered PM options, they will not have to chance to adopt it. Questions about participants’ immigration status were not part of the demographic questionnaire and the focus groups discussion guide, but some participants shared their immigration status as undocumented in the discussions, and other participants assented that they were undocumented in the U.S. Another barrier mentioned by participants was distrust of the government keeping or having access to their genetic information because the government could share their information with immigration enforcement agencies, such as the U.S. Immigration and Customs Enforcement Agency (ICE), known by participants as “the *migra*,” and deport them.

**Table 3:** Barriers to Dissemination and Adoption of Precision Medicine Approaches

Barriers		
Lack of Health Insurance	• “I wonder how many people could have access [to PM] benefits because many of us do not have “aseguranza” [health insurance], at least I do not have it.”	• “… people without [immigration] papers cannot buy health insurance and do not qualify for government benefits.”
Financial Burden	• “… [PM] is an innovation, and it is something that everyone would like to have access to, but the negative aspect [of it] is that it must be expensive, so people would need to be in debt.” • “… [PM] would not benefit the Hispanic community because it is a segmented community, and like others mentioned before me, I think that the cost of [PM] is a barrier.”	• “The cost that [a person] might have to incur because a test for each gene is needed … it is something that everyone wishes to be part of, but money is the problem.”
Immigration Status	• “I think that it is an ambitious program … in general, a large segment of Hispanics without papers could not access [PM], and it would not be inclusive.” • “… a large number of Hispanics without papers would not benefit because undocumented immigrants do not have access to many programs.”	• “I think that one’s immigration status is a problem because we are not protected. If something bad occurs, who is going to defend us?”
Distrust of Government	• “… if the government keeps our [genetic] information, it can use it to deport us.”	• “I am worried that the government can have access my [genetic] information because it could use it for experiments without my knowledge and authorization.”
Limited English Proficiency	• “I think that because Hispanics do not speak English, they do not go to the hospital, that is why it is important to have [there] someone who speak Spanish.”	• “I might not like to use it [PM] because there is no information about precision medicine in Spanish, but once they learn what [precision] medicine is, I think that the majority would like to get it.”
Low Literacy	• “For me, everything that I am learning about genes and PM is not easy to understand because I did not complete secondary education, everything is new and am just learning about [PM] because I had never heard about all of this.”	• “I think that it is possible [to disseminate PM] through all means available that exist today, but the way that it is told should be changed. We know that the technical part would be term precision medicine o personalized medicine, so many would be confused when they hear these words … how about calling it preventive medicine so tha it could be easy to understand.”
Cultural Norms	• “in Latin America, doing new things are like a taboo. When people need to donate blood … people are afraid to donate blood or donate organs.”	• “People will have doubts about [PM] because in the Hispanic culture, we will not participate in something unless we see or hear about someone who is cured with this medicine.”
Fear about Genetic Testing Results	• “The fear that a person carries a gene; for example, my grandfather died from cancer, and now my sisters and I are afraid that we inherited his genes. That is why we are afraid of the results … even though we could know early if we can develop cancer, it is scary.”	• “The fear that a person carries a gene; for example, my grandfather died from cancer, and now my sisters and I are afraid that we inherited his genes. That is why we are afraid of the results … even though we could know early if we can develop cancer, it is scary.”
Lack of Transportation	• “I think that [a barrier] might be the location where [PM] is offered. There are people who cannot get to their appointments due to lack of transportation.”	• “Many of Hispanic families cannot have money to buy a car and other stuff, and I think that even if they would want this medicine, they could not go [there] even if they want to get precision medicine.”
Newness of PM Approach	• “… number 3 mentioned that from now on, medicine will be more efficient and faster, [but] that no one knows with exactitude …”	• “The problem [for me] will be if I am going to be one of the first ones participating in [PM], I will not know if it will be effective or not.”
Lack of Information about Precision Medicine	• “The lack of information about precision medicine or personalized could be one of the first barriers, the second reason is the lack of information about the type of treatments will offer.”	• “I might not use [PM] because there is no information about it, but once there is information in Spanish, I could learn more about it, and I might use [it].”

Participants mentioned their limited English proficiency as a barrier because, if they could not communicate with their English-speaking doctors, they could not learn and ask about PM. In addition, participants’ low literacy was mentioned as a barrier due to the high literacy level of PM medical jargon, which is unfamiliar to lay people. Other barriers mentioned by participants were cultural norms discouraging the adoption of unfamiliar procedures in one’s healthcare, fear about genetic testing results that could show a genetic variation for predisposition to develop a serious disease, lack of transportation to go to places where genetic testing is offered, newness of PM approaches because participants were not aware of any case in which patients benefited from them. and lack of information about PM because participants had no knowledge and never heard about this approach in medicine prior to the focus group session.

### Facilitators to dissemination and adoption of precision medicine approaches

Figure 2 summarizes the themes that emerged related to facilitators to dissemination and adoption of PM, and sample quotes are listed in Table 4. PM is a novel approach in medicine that Hispanics/Latinos had no knowledge of and had never heard about. Consequently, participants were asked during the focus groups to share what could facilitate learning more about PM approaches and adopting the testing that these approaches requires. Table 4 lists the facilitators that emerged as themes. Familie***s***, according to most participants, are extremely important in healthcare decision making and would need to be consulted before having a PM genetic test or treatment. Married participants would consult with their spouses and children in a family meeting before adopting PM. The decision would be a family matter, and everyone would understand the implications of the decision. Participants who were single mentioned that they would consult with their parents and siblings first, then with members of their extended family. Educational material in plain language, with pictures or graphics in Spanish about PM, and assistance programs for the uninsured were mentioned as potential facilitators to the dissemination and adoption of PM.

**Table 4:** Facilitators to Dissemination and Adoption of Precision Medicine Approaches

Facilitators		
Family	• “My family, especially my daughters, help me make health decisions. If they tell me that I need to do this medicine, I will do it.”	• “Hispanics have large extended families and that before to make decisions, we ask our sisters, mothers, even our friends before we make any health decision.”
Information in Spanish	• “The majority of Hispanics living here do not speak English at all, so if the information [about PM] is in our language, it may help us understand because it is in Spanish.”	• “I think that the doctor who speaks the same language I speak [Spanish] can help me understand. If the doctor speaks English I would not get a [genetic] test.”
Plain Language and Graphics	• “When [PM] information is distributed in pamphlets, it should not have to many written words. It should have drawings so that everyone can understand. For example, I only finish elementary school …”	• “Most of the time, the information about new things like this medicine is not given in a way that people can understand. Then little by little [doctors] can explain to us what diseases [PM] could treat and cure.”
Assistance Programs for Uninsured	• “I think that free programs that can offer free genetic testing would be good for those who do not have health insurance.”	• “I do not go to visit the doctor becasuse I do not have health insurance. I wonder who will help me to go to the doctor to get a genetic test. Who will help me if I need to go back to the doctor again?”
Trust in Doctors and Healthcare staff	• “… doctors are accredited and have all the support they need for this type of medicine, so patients can say ok …” • “… a doctor studied medicine and in [his/her] office [he/she] has a diploma. That makes me more confident.” • “If the doctor tells me that I have a chronic disease and need to use PM, I will use it.”	• “Nurses who work with doctors study to work in a hospital, and they have to be good to help doctors. I trust them.” “… since this medicine’s focus is precision, I trust a doctor because [he/she] looks into each case, each patient.”
Trust in Well-known Hospitals	• “I feel good when I get health information at the hospital because it is the place where I get healthcare.” • “… but that information must be in Spanish though.”	• “I would trust more if it comes from a hospital because there, there is the appropriate equipment. I fully trust if the [PM information] comes from a hospital.”
Trust in Academic Institutions	• “If universities like Vanderbilt will keep genetic information, I can trust my information … they do not give our medical information to the government”	• “I heard that universities do not do research unless one gives them permission. I guess it would be the same with precision medicine.”
Source of Information: Healthcare Provider	• “… a doctor can give accurate information, so that people can express real interest, that is why they are professional.”	• “I would not trust anyone but doctors because they can explain to us very well that it is to detect a disease in my blood.”
Source of Information: Community Organizations	• “.. the best way to teach or explain to people about [precision] medicine it would be through foundations, hospitals, churches, even at schools could be explained how this medicine works and how will benefit people.”	• “I think that if a place like this [community center] offers presentations, forums, workshops, and other things, people can understand better what precision medicine is.”
Personal Motivation	• “It would be up to each person’s interest. If someone has the interest in being cured, [he/she] will use precision medicine.”	• “Anyway, persons who are already sick will use the service [precision medicine]. I think that if a person feels healthy will not.”
Altruism (societal benefit)	• “Yes, I would participate in [precision medicine] because [it] would help to act fast to treat a disease to help others in the future”	• “.. if the doctor asks me to provide my genetic information for future researches, I believe that like human beings, [we] have to accept the [genetic] test because it is for the benefit of the advance of medicine for other people …”

Participants expressed trust in their doctors regarding the dissemination and adoption of PM approaches due to their academic training and medical licensure. Participants also expressed trust in nurses and other healthcare providers because they work with physicians. All participants agreed that they would prefer a doctor who speaks their language to help them better understand about PM and to answer questions about PM. Participants would consider PM information reliable if it comes from well-known hospitals. If PM information were presented in a lesser-known health hospital, participants expressed they would have lower trust in the source. Participants expressed confidence that academic institutions could be trusted to keep their genetic information because they believed these institutions would not share their genetic information without consent. As for the source of information about PM, participants mentioned that doctors, foundations, hospital, churches, and schools are the most trusted sources. They also identified individual, familial, and societal benefits as PM facilitators. For example, a PM-based treatment based on a pharmacogenomic test may be more effective for an individual patient than the traditional treatment approach. If a genetic variation for cancer genetic predisposition is identified, the individual’s family could seek to get a genetic test to find if someone else in the family carries the same genetic variation. Some mentioned that PM approaches would generally benefit society, even if it did not help every patient.

## Discussion

Advances in genetics and genetic testing in the last 20 years have brought PM approaches to the forefront in the conversation about innovations in medicine, pointing to the need to examine the dissemination and adoption of PM approaches to traditionally underserved groups [25]. In the current qualitative study, most Hispanics/Latinos had heard about genes from formal sources of information, such as schools, and they generally understood what they were, although not in detail. However, the term genetics was not as familiar as genes; consequently, participants often used the terms genes and genetics interchangeably. Finally, participants across the focus groups had never heard of and had no knowledge about PM. This corroborates previous work that has identified lack of public knowledge and awareness as the main challenge to dissemination and adoption of this new healthcare approach [26].

### Barriers

Across the focus groups, financial burden was mentioned as a barrier to dissemination and adoption of PM approaches because over two thirds (68%) of the focus group participants had a monthly income of $2999 or less. In underserved populations such as Hispanics/Latinos in the US, poverty limits access to and information about health care [27]. Participants agreed that NIH’s definitions of genes and genetics were short and that they were too technical and written at a high literacy level. This perception could be a function of the low levels of education in our participants. Over a third of participants (34.1%) in this study had 8 years of education attainment in addition to being low income. Similarly, a study in California found that Hispanic/Latino immigrants living in poverty had low levels of literacy due to their low educational attainment [28]. More than half of participants (53.6%) had limited English proficiency knowing little English or nothing at all. Consequently, Hispanics/Latinos continue facing acculturation obstacles due to their limited English proficiency and cultural sensibility to innovations in healthcare [29]. Therefore, participants mentioned that these definitions needed to be culturally, linguistically, and literacy appropriate for other community members with low levels of literacy and limited English proficiency to understand information in order to make informed PM-related decisions.

A majority of our focus group participants (82.0%) was uninsured. People without insurance face similar barriers to accessing the one-size-fits-all healthcare approach. This is consistent with previous research on Hispanics/Latinos’ barriers to healthcare [30]. Previous research conducted by Karoly and colleague (2011) found that undocumented immigrant Hispanic/Latino parents with U.S. born-children did not enroll their children in government-offered programs. Parents’ undocumented immigration status was the determinant factor to distrust government. Focus group participants in this study expressed distrust of government keeping their genetic information because it can have access to them and share them with immigration enforcement institutions, such as the U.S. Immigration and Customs Enforcement Agency (ICE), to deport those who are undocumented immigrants [31]. The intersection of poverty with race, ethnicity, and immigration status demonstrates that being low income leads to negative health outcomes [27].

Disseminating the provisions of the Genetic Information Nondiscrimination Act of 2008 (GINA) [32] is imperative to address this issue of mistrust. Inadequate understanding of PM may hinder the participation of Hispanics/Latinos in the seminal “All of Us” (AoU) Research Project led by NIH. AoU seeks to gather data from at least one million diverse people living in the U.S. to study the interactions among genetic makeup, environment, and lifestyle and their relation to health outcomes and disparities [33].

Focus group participants mentioned that their cultural norms were barriers to PM adoption. Hispanics/Latinos are reluctant to adopt innovations, particularly in healthcare, due to their experiences of having been diagnosed and treated using the one-size-fits all healthcare paradigm. Different terms used for new healthcare approach, such as precision medicine and individualized/personalized medicine create concern, confusion, and fear among people in marginalized socioeconomic and cultural groups [34]. Fear about the results of genetic testing was another barrier mentioned by participants due to the uncertainty created by these findings. The fear is not only limited to the disease, but to the financial burden, access to care, and potential consequences of loss of privacy. Fear of results was also reported in a study on gender differences in genetic testing results among Latinos [35]. Lack of transportation was reported as an additional barrier to the dissemination and adoption to PM. Latino caregivers of children with complex medical conditions face transportation challenges [36]. In addition to financial burden of buying a car, immigration policies in Tennessee, which do not allow undocumented immigrants to obtain drivers’ license, limit the ability to seek care due to the risk of being arrested and deported [37].

Participants expressed concern about the newness and lack of information about PM. A study found that Spanish-speaking parents’ barriers to initiate and comply with human papilloma virus (HPV) vaccine were mostly based on their perceived newness and lack of information about the benefits and risk of it [38]. Participants expressed the same concern about PM as a new approach in healthcare. Hispanics/Latinos’ uneven knowledge and awareness of the terms genes, genetics, and PM require focused attention because this population is also underrepresented in genetic associations studies [39]. Current studies have focused on dissemination and adoption of innovation among non-Hispanic Whites, while neglecting Hispanics/Latinos [13]; consequently, there is a risk of exacerbating health disparities in this hard-to- reach population [40]. The Hispanic/Latino population is diverse with different social, cultural, and linguistic backgrounds, which may include low or complete absence of English proficiency, lacks or has limited access to healthcare, has low or lacks educational attainment, lives in poverty and marginalization, and experiences housing insecurity [41–45].

### Facilitators

In terms of facilitators to dissemination and adoption of PM approaches, this study was consistent with previous research in emphasizing the importance of the family in Hispanic/Latino cultures as the source of support, responsibility, and respect when health decisions are made [46]. Similar to Crooke et al. (2016) study on healthy eating, Hispanic/Latino nuclear families play an important role as a social network. The family network provides the necessary social support when a health-related decision is made [47]. Information in Spanish about PM that uses succinct plain language with graphics, pictures, and drawings in printed educational material best explains what PM is and what the benefits of this approach in healthcare are. The more knowledge Hispanics/Latinos have about PM, the more chances they have to positively engage with dissemination and adoption of this innovation [14, 48].

Participants mentioned that assistance programs for uninsured may help them to adopt PM approaches. Assistance to get genetic testing or treatment may be provided at Federally Qualified Healthcare Centers, which are prohibited to discriminate against national origin under Title VI of the Civil Rights Act. Providing equal access to health care will help ameliorate Hispanics/Latinos’ disadvantage due to socioeconomic characteristics, such as income, immigration status, and language [49, 50]. Trust in doctors and healthcare staff was important for the physician-patient relationship. The dissemination and adoption of PM relies on fostering these trusting relationships. Participants in this study generally expressed trust in doctors in general, even if they did not speak Spanish, in contrast to previous survey studies that have found higher levels of physician distrust Hispanics/Latinos’ compared to non-Hispanic Whites [51]. This inconsistency may be due in part to observed geographic variation in levels of Hispanic/Latino physician distrust [52], as well as differences in methodology across studies [53]. They also expressed trust in in well-known hospitals. It is important to continue fostering patient-centered care that includes patients’ input in health-related decisions and offers the opportunity to establish communication, trust, and rapport between doctors and patients leading to positive patient care experience [54]. Participants mentioned that they trust in academic institutions to keep their genetic information and health history. Academic institutions can earn patients trust by establishing community-academic partnership with community organizations focused on CEnR to allow a bi-directional relationship in all the stages of research [20]. Additionally, participants mentioned that they would trust the information about PM shared in churches and community centers if the sources of information come from physicians and academic institutions. Dissemination information about PM through these trusted channels could help address barriers stemming from distrust in government.

Similar to Hamilton et al. (2016), personal motivation was a facilitator because PM may offer individual benefits such as getting a genetic test to learn if a person carries a specific genetic trait and using the test to identify the most effective treatment [35]. The focus group participants also mentioned altruism as the main reason to support the dissemination and adoption of PM for societal and scientific benefit, even if they did not benefit themselves, a finding found in another study [55].

### Limitations

The purposive sampling used for the recruitment of participants for the focus groups is a limitation of qualitative study designs. Consequently, the sample of this study may not be representative of the Hispanic/Latino community in Nashville or the broader U.S. population. Furthermore, since the sample in this study did not include non-Hispanic Whites and non-Hispanic Blacks, we cannot determine if the findings are unique to Hispanics/Latinos versus other non-Hispanic groups. Additionally, in this study it was not feasible to tie barriers and facilitators to actual uptake and use of PM. Future implementation research may benefit from measuring the barriers and facilitators identified here to determine their relevance to decision making in the healthcare context. Finally, this study focused on patient-level barriers and facilitators, though identifying and addressing barriers at the provider-hospital-and system- levels is also needed.

### Implications for public health practice and research

The transformational character of PM presents a challenge to its dissemination and adoption among socially disadvantaged minorities in general and Hispanics/Latinos in particular, thus hindering the potential benefits of PM breakthroughs [56]. Educational health materials developed without the engagement of community members are often ineffective and can unintentionally contribute to widening health disparities in the dissemination and adoption of innovation in healthcare [57]. These findings highlight the need to include Hispanics/Latinos as stakeholders in research and discussions regarding the development of effective PM educational materials, rather than excluding them due to their lack of training and experience as researchers [58] . In addition, healthcare institutions should include genetic counselors who are trained in public health and cultural and linguistic competency to serve diverse patient populations. Genetic counselors can play a crucial role as health educators to help translate the technical terms used in PM to lay language.

## Conclusion

The current study highlights a striking need for improved dissemination of information about PM to Hispanics/Latinos. It also identifies a number of barriers and facilitators to PM adoption to guide the direction of future implementation research. This study contributes to the existing body of literature on PM. By exploring the knowledge, awareness, barriers, and facilitator to implementation and adoption to PM, as a new healthcare paradigm among Hispanics/Latinos. This qualitative research also offers important insights for the “All of Us” national research program, which aims to enroll one million diverse participants, including Hispanics/Latinos, to reflect the diversity of the population in the U.S. Furthermore, the findings of this study can be used to create education materials that are culturally-and- linguistically appropriate for low literacy levels in English and Spanish about PM. Additional research is needed to explore the interaction between these patient-level barriers and facilitators with other provider and system-level barriers and facilitators to PM dissemination and adoption.

## Data Availability

The data used and analyzed during the current study are available from corresponding author on reasonable request.

## Abbreviations

PM: Precision medicine
U.S.: United States
CenR: Community-engaged research
PCC: Progreso Community Center
MMC: Meharry Medical College
CITI Program: Collaborative Institutional Training Initiative
ID (number assigned to participants in focus groups): Identification Document
NIH: National Institutes of Health
Nvivo: Qualitative software version 11.4.3
SPSS: Statistical Package for the Social Science
ICE: U.S. Immigration and Customs Enforcement Agency
GINA: Genetic Information Nondiscrimination Act of 2008
AoU: “All of Us” Research Project.
HPV: Human papillomavirus
